# Explainable AI in medical imaging: an interpretable and collaborative federated learning model for brain tumor classification

**DOI:** 10.3389/fonc.2025.1535478

**Published:** 2025-02-27

**Authors:** Qurat-ul-ain Mastoi, Shahid Latif, Sarfraz Brohi, Jawad Ahmad, Abdulmajeed Alqhatani, Mohammed S. Alshehri, Alanoud Al Mazroa, Rahmat Ullah

**Affiliations:** ^1^ School of Computing and Creative Technologies, University of the West of England Bristol, Bristol, United Kingdom; ^2^ Cybersecurity Center, Prince Mohammad Bin Fahd University, Alkhobar, Saudi Arabia; ^3^ Department of Information Systems, College of Computer Science and Information Systems, Najran University, Najran, Saudi Arabia; ^4^ Department of Computer Science, College of Computer Science and Information Systems, Najran University, Najran, Saudi Arabia; ^5^ Department of Information Systems, College of Computer and Information Sciences, Princess Nourah bint Abdulrahman University (PNU), Riyadh, Saudi Arabia; ^6^ School of Computer Science and Electronic Engineering, University of Essex, Colchester, United Kingdom

**Keywords:** explainable AI, federated learning, brain tumors, GoogLeNet, medical diagnosis

## Abstract

**Introduction:**

A brain tumor is a collection of abnormal cells in the brain that can become life-threatening due to its ability to spread. Therefore, a prompt and meticulous classification of the brain tumor is an essential element in healthcare care. Magnetic Resonance Imaging (MRI) is the central resource for producing high-quality images of soft tissue and is considered the principal technology for diagnosing brain tumors. Recently, computer vision techniques such as deep learning (DL) have played an important role in the classification of brain tumors, most of which use traditional centralized classification models, which face significant challenges due to the insufficient availability of diverse and representative datasets and exacerbate the difficulties in obtaining a transparent model. This study proposes a collaborative federated learning model (CFLM) with explainable artificial intelligence (XAI) to mitigate existing problems using state-of-the-art methods.

**Methods:**

The proposed method addresses four class classification problems to identify glioma, meningioma, no tumor, and pituitary tumors. We have integrated GoogLeNet with a federated learning (FL) framework to facilitate collaborative learning on multiple devices to maintain the privacy of sensitive information locally. Moreover, this study also focuses on the interpretability to make the model transparent using Gradient-weighted class activation mapping (Grad-CAM) and saliency map visualizations.

**Results:**

In total, 10 clients were selected for the proposed model with 50 communication rounds, each with decentralized local datasets for training. The proposed approach achieves 94% classification accuracy. Moreover, we incorporate Grad-CAM with heat maps and saliency maps to offer interpretability and meaningful graphical interpretations for healthcare specialists.

**Conclusion:**

This study outlines an efficient and interpretable model for brain tumor classification by introducing an integrated technique using FL with GoogLeNet architecture. The proposed framework has great potential to improve brain tumor classification to make them more reliable and transparent for clinical use.

## 1 Introduction

The brain serves as the main component of the body which is responsible for controlling cognitive and regulating physiological functions in the body. Brain tumors are one of the common diseases with a severe impact on quality of life (29, 30). The early diagnosis of brain tumors can improve patient outcomes and increase their chances of survival. In the medical imaging field, MRI provides the potential help in efficiently diagnosing the brain tumor by providing a clear picture of the cerebral lesions in the patient’s brain. The precise classification of brain tumors using MRI images can play a vital role in supporting treatment decisions and improving patient survival outcomes (1).

DL has recently emerged as a transformative approach to automatic brain tumor classification. It uses huge datasets and complex neural network architecture to detect subtle indicators of malignancy in MRI scans (2). Although conventional DL approaches are generally effective, they usually require centralized data aggregation and raise concerns about data privacy and security due to the uneven data distribution among participants (3). FL is a new paradigm that allows various participants to jointly train models without breaching the privacy of sensitive information about patients (4). FL facilitates the training of algorithms directly from the local datasets and improves data security. The decentralized nature of the FL approach addresses privacy issues. It enforces model resilience by incorporating a larger variety of data, which is crucial for developing a more robust and generalized model in medical image analysis (5). Despite the several benefits of traditional FL models, there is a lack of model interpretability, which is an important factor in the healthcare domain where sensitive data are involved. The black-box nature of FL models makes it difficult for clinicians to grasp the decision-making process, potentially reducing trust and hindering their practical implementation (6). To facilitate the clinical decision-making process in the healthcare system, it is crucial to provide clear explanations. Explainable federated learning (XFL) has been developed to provide a solution to the challenges related to the privacy and security of sensitive information such as patients. The integration of XAI techniques with XFL’s privacy and decentralization advantages results in a more comprehensive and provide more interpretable insight in the decision-making process.

This article introduces an effective FL scheme for brain tumor classification utilizing MRI image fusion with an explainable framework that addresses the challenges of model interpretability. GoogLeNet provides numerous benefits compared to the other pre-trained CNN models. It integrates various filter sizes, which improves its ability to extract detailed features from MRI images. The key motivation behind the integration of GoogLeNet with the FL framework is its high accuracy and efficiency, which is important to handle medical imaging data. Despite their usefulness, the opaque nature of classification models introduces significant challenges in a clinical environment. This problem can significantly undermine trust in AI implementations that rely on interpretability. To ensure trust is maintained, transparency is paramount in healthcare care, which helps to ensure a clear understanding of the reasoning behind each prediction. To enhance the model transparency and interpretability, the proposed architecture utilizes Grad-CAM and Saliency map visualizations.

The key contributions of this study are summarized in the following.

1. This article develops an explainable FL framework that facilitates collaborative model training without exposing sensitive patient information. This model strengthens the generalization, interpretability, and robustness of the model that address the privacy and security limitations of traditional DL models.
2. The proposed framework integrates a pre-trained GoogLeNet architecture as a core classifier within the FL framework. GoogLeNet exhibits superior classification performance compared to the other pre-trained CNN models in decentralized environments. Furthermore, the inclusion of Grad-CAM and Saliency Map visualization further enhances the model’s transparency by offering visual insights for its prediction process.
3. An efficient aggregation mechanism integrates the contributions of multiple participants to improve accuracy and robustness, tame data heterogeneity, and address bias issues. Meanwhile, Grad-CAM and Saliency maps provide transparency to ensure a decision-making process is transparent and trustworthy for clinical applications.

The remainder of the article is organized as follows. Section 2 summarizes some latest state-of-the-art research contributions related to FL-based brain tumor classification. Section 3 elaborates on the mathematical model and detailed workflow of the proposed architecture. Section 4 presents a brief discussion of the experimental outcomes and their implications. Finally, Section 5 concludes the research by summarizing key insights.

## 2 Related work

Medical imaging serves as an important tool to identify, diagnose, and classify brain tumors at an early stage (7). MRI, computed tomography (CT), and positron emission tomography (PET) are commonly used techniques that enable clinicians to visualize brain structures and assist in accurate detection of brain abnormalities. MRI is the most commonly used technique due to its high contrast resolution, which allows detailed observation of brain tissues without ionizing radiation. The precise classification of the brain tumor plays an important role in deciding the right treatment plan for the patient. The importance of medical imaging in tumor classification lies in its ability to provide non-invasive, detailed, and reproducible visual data. Advanced image processing techniques such as convolutional neural networks (CNNs) have been widely investigated to improve tumor classification, improve accuracy, and reduce radiologists’ dependence on manual interpretation.

Several studies in the existing literature proposed advanced DL methods for an accurate classification of brain tumors. Chatterjee et al. (8) proposed DL models ResNet (2 + 1) D and ResNet Mixed Convolution for brain tumor classification. The authors claimed that these models outperformed traditional 3D convolutional models by learning spatial and temporal relationships more effectively, achieving the best test accuracy of 96.98% with the ResNet Mixed Convolution model. Another DL-based approach for brain tumor classification proposed by (9) involved two stages: segmenting brain tumors from multimodal magnetic resonance images (mMRI) and classifying tumors using the results of the segmentation. Using a 3D deep neural network, the method achieved a dice score of 0.749 and an F1 score of 0.764 on validation data. A method utilizing CNN and a genetic algorithm (GA) was proposed by (10) for the non-invasive classification of different glioma grades using MRI, achieving 94.2% accuracy. A fully automated DL-based approach for the multi-classification of brain tumors was proposed by (11). The authors incorporated CNNs with hyperparameters optimized through grid search and achieved an accuracy of 92.66% in brain tumor detection. Other notable approaches in the literature highlight several prominent DL modalities utilized for brain tumor classification. These include the HCNN ensemble CRF-RRNN (12), ensembles such as 3D-CNN combined with U-Net (13).

FL has recently emerged as a promising technique for brain tumor classification. Several studies have explored the application of FL in the classification of brain tumors using MRI (11). proposed a privacy preserving FL architecture for brain tumor classification. Similarly, Sheller et al. (14) demonstrated the effectiveness of FL in brain tumor segmentation through multi-institutional collaboration, achieving 85.1% accuracy. Tedeschini et al. (15) proposed an FL scheme for cancer diagnosis using the message queueing telemetry transport (MQTT) protocol to address performance issues in geographically distributed systems, achieving 87.4% accuracy. Islam et al. (16) proposed an FL approach combined with a CNN ensemble architecture for the detection of brain tumors. Their method also addressed the challenges associated with centralized data collection, achieving an accuracy of 91.05% using the CNN-based FL framework.

The existing literature also presents some remarkable and novel studies on XAI-based brain tumor classification frameworks. For example (17), integrated attention Maps, SHAP, and LIME methods with the hybrid Vision Transformer (ViT) and Gated Recurrent Unit (GRU) to improve the interpretability of classified MRI scans. Kumar et al. (18) proposed the Subtractive Spatial Lightweight (SSLW) CNN for brain tumor classification, emphasizing its efficiency in reducing computational time while achieving an accuracy of 80.15%. The study also incorporated XAI techniques, particularly Class Activation Mapping (CAM), to improve the transparency and interpretability of the model. CAM demonstrated a strong alignment with human decision-making, achieving a visual match rate of 86%–95%. Although the results of the AI models in existing studies are quite encouraging, the research highlights the need to further improve XAI techniques and model optimization to enhance accuracy and wider use of models in clinical settings.

In applications of brain tumor classification, existing FL-based studies exhibit few potential issues such as limited interpretability, lower model performance due to data heterogeneity, and the use of centralized data, which make them vulnerable to privacy concerns. Although some studies have addressed these issues by incorporating explainability within FL frameworks, they often lack advanced visualization methods to provide meaningful insights into the model’s decision-making process. The proposed framework addresses these limitations by integrating the GoogLeNet architecture within an XFL setting. GoogLeNet, known for its deep network with Inception modules, offers efficient feature extraction and classification capabilities, making it well-suited for complex medical imaging tasks such as brain tumor classification.

To enhance model interpretability, we incorporate Grad-CAM and Saliency Map visualization techniques. Grad-CAM highlights the important regions in the input image that contribute to the prediction of the model, allowing clinicians to understand which areas the model focuses on when classifying a tumor. Saliency maps provide another layer of explainability by showing the pixel-level importance, offering a finer granularity of interpretation. The use of these visualization methods brings greater transparency, allowing healthcare professionals to verify the model’s results and have confidence in its decision-making process, which is very important in several healthcare applications.

## 3 The proposed architecture

**Figure 1:**
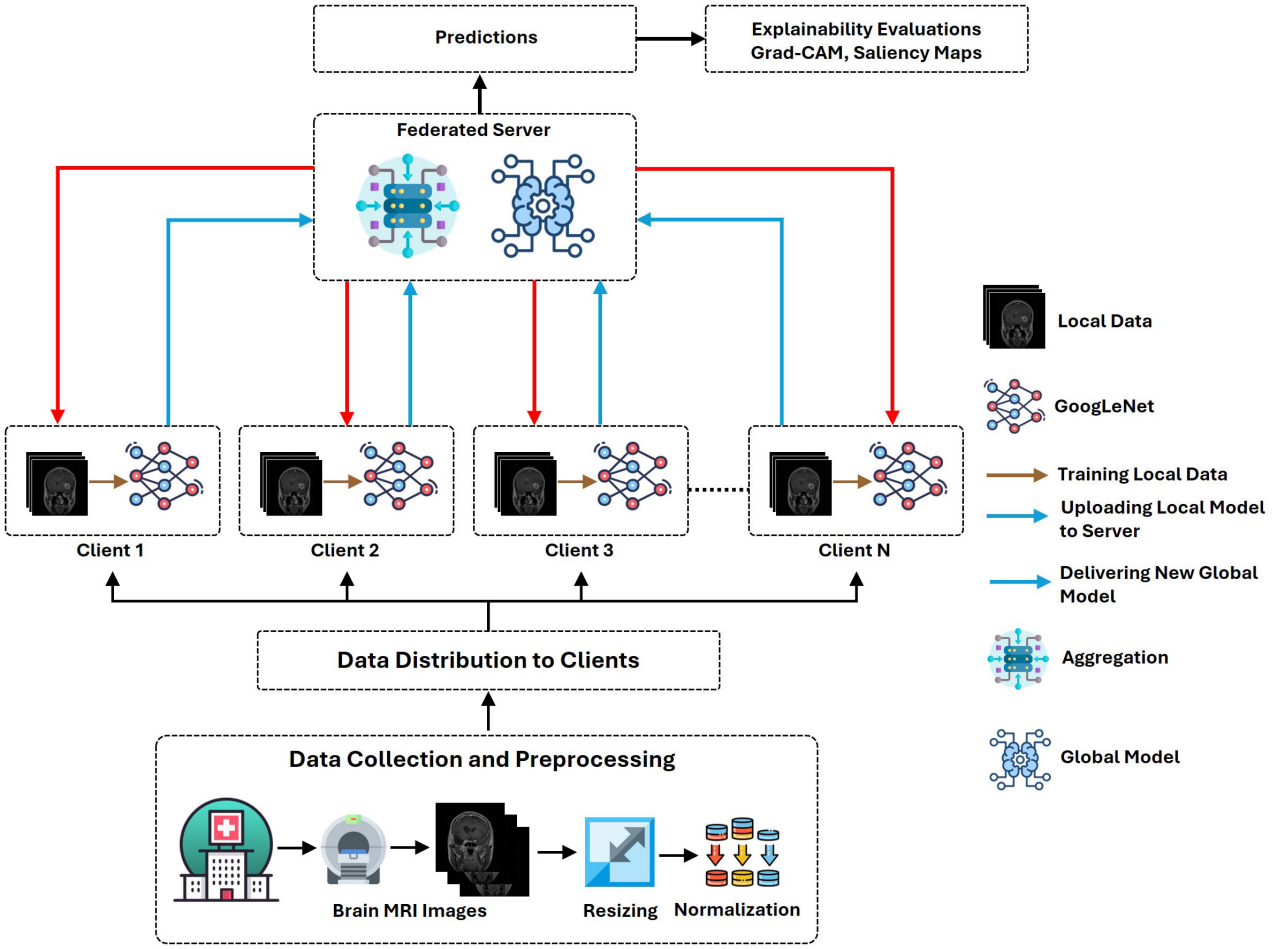
Workflow illustration of the proposed XFL model for classifying brain tumors.

This proposed architecture utilizes the “Brain Tumor MRI Dataset” for training and performance evaluation. This dataset is open source and publicly accessible for use on the Kaggle platform (19). The dataset, which comprises 7023 human brain MRI images, is divided into four categories: glioma, meningioma, no tumor, and pituitary. This dataset offers a valuable platform for the bridge between artificial intelligence and medical imaging to showcase practical applications for brain tumor classification. **Figure 1** presents the operational flow of the proposed architecture.

### 3.1 Data preprocessing and distribution

Data preprocessing is an important stage in ML to ensure data quality and consistency (20). It involves multiple operations that improve model performance, extract meaningful insights from the data, and build accurate and reliable models.

#### 3.1.1 Dataset definition

**Figure 2:**
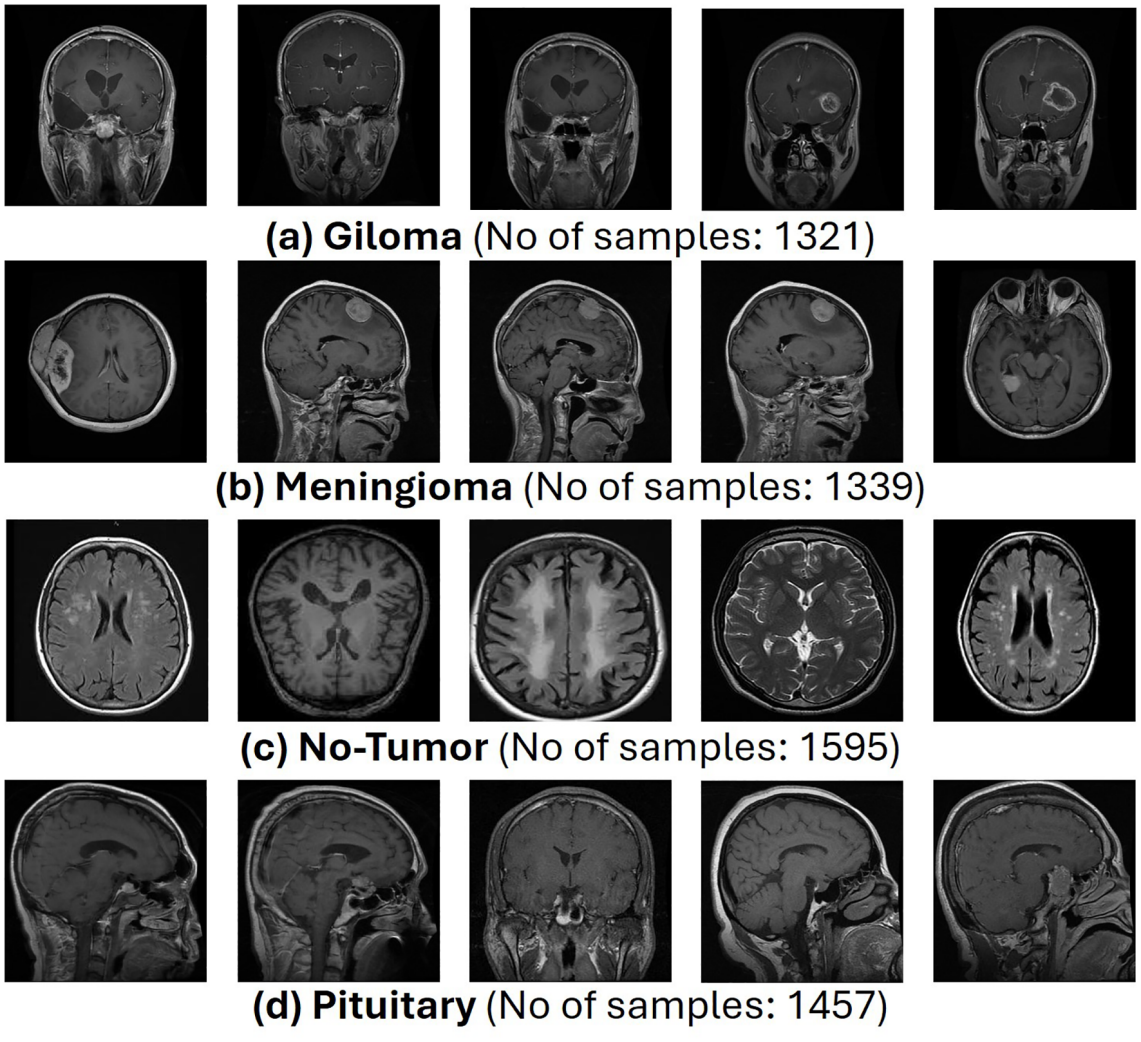
Representative samples from brain tumor MRI dataset.

The complete dataset, referred to as *D*, is divided into two Google Drive directories, namely, train and test each directory containing images paired with their labels. Representative samples from each dataset class are presented in **Figure 2**.

#### 3.1.2 Dataset transformation

Each image *x* in the dataset undergoes a series of transformations that prepare it for input into the model.

1. **Resizing:** Each image *x* is resized to a fixed dimension (*H,W*). In this case, the resizing operation is defined by Equation 1:
(1) $$x^{\prime }=\text{Resize }(x,\left(H,W\right))$$
  where *H* = 224 and *W* = 224. This standardizes the input size, which is essential for consistency in model training.
2. **Normalization and Conversion to Tensor:** In the preprocessing pipeline, the resized image *x*′ is converted into a tensor and normalized, as shown in Equation 2.
(2) $$x^{\prime \prime }=\text{ToTensor }\left(x^{\prime }\right)$$

This step maps pixel values to a normalized range (typically between 0 and 1 or −1 and 1), making the data suitable for neural networks that are sensitive to input scales. Let *T* represent the complete transformation function, including resizing and tensor conversion. The transformation applied to each image *x* can be represented by Equation 3.


(3) $$x^{\prime \prime }=T\left(x\right)=\text{ToTensor }(\text{Resize }(x,\left(H,W\right)))$$

This equation succinctly captures the entire pre-processing pipeline for each image.

### 3.2 Data distribution to clients

After preprocessing, the dataset *D* is divided among *N* clients, each receiving a disjoint subset for training. The goal is to partition the dataset into *N* subsets {*D*_1_ *,D*_2_ *,…,D_N_*}, with each subset *D_i_* assigned to a specific client.

#### 3.2.1 Splitting the dataset

The dataset *D* is partitioned into subsets as described in Equation 4.


(4) $$D=\overset{N}{\underset{i=1}{\cup }}D_{i}\text{ and }D_{i}\cap D_{j}=\emptyset \;\text{for}\;i\neq j$$

The size of each subset $\left|D_{i}\right|$ is determined by the total dataset size |D| and the number of clients N . For the first N−1 clients, the size of each subset is approximately as shown in Equation 5.


(5) $$\left|D_{N}\right|=\left|D\right|-\sum_{i=1}^{N-1}\left|D_{i}\right|$$

For the last client, the subset size accounts for any remaining to ensure that all data are distributed.

### 3.3 Data loading to client-specific loaders

Each client *i* uses a data loader to handle its specific subset *D_i_*. The data loader is responsible for batching the data and feeding them into the model during training.

#### 3.3.1 Batching

Each dataset *D_i_* is divided into batches of size *B* as shown in Equation 6.


(6) $$D_{i}=\left\{B_{i,1},B_{i,2},\ldots ,B_{i,K_{i}}\right\}$$

where $K_{i}=\frac{\left|D_{i}\right|}{B}$ is the number of batches for the client i . Each batch $B_{i,j}$ contains a set of input-output pairs (x,y) , as shown in Equation 7.


(7) $$B_{i,j}=\left\{\left(x_{j,1},y_{j,1}\right),\ldots ,\left(x_{j,B},y_{j,B}\right)\right\}$$

with each batch $B_{i,j}$ having a size of B .

#### 3.3.2 Data loader

The data loader *L_i_* for the client *i* iterates over the batches and sends them to the model for training, as shown in Equation 8.


(8) $$L_{i}:D_{i}\to \left\{B_{i,1},B_{i,2},\ldots ,B_{i,K_{i}}\right\}$$

This function facilitates the sequential loading of data in manageable portions (batches), optimizing memory usage, and training efficiency.

### 3.4 Client-side training with GoogLeNet

Each client *i* performs local training using the GoogLeNet architecture. The model components and their functionalities are briefly described in the following.

#### 3.4.1 Convolutional layers

Convolution extracts spatial features by applying filters to the input image or feature maps. Each filter learns a specific pattern, such as edges or textures. In addition, the ReLU activation function introduces non-linearity, which allows the network to learn more complex features. Finally, pooling reduces the spatial dimensions, decreasing computation while preserving the most significant features. The detailed operation of these layers is described below.

**1. First Convolution Layer:** Due to the large filter size and stride, the model captures large-scale features from the input image, as described in Equation 9.


(9) $$X_{1}=\text{ReLU }\left(W_{1}*X+b_{1}\right)$$

- Filters: *W*_1_ with shape 64 × 7 × 7 × 3 (64 filters of size 7 × 7 with 3 channels).
- Stride: 2, Padding: ‘valid’ (no padding).
- Output Shape: $X_{1}\in {\mathbb{R}}^{109\times 109\times 64}$ .

**2. First Max-Pooling Layer:** It reduces spatial dimensions, providing translation invariance defined by Equation 10.


(10) $$X_{2}=\text{MaxPool }\left(X_{1},\left(3,3\right),\text{ stride }=2\right)$$

- Output Shape: $X_{2}\in {\mathbb{R}}^{54\times 54\times 64}$ .

**3. Second Convolution Layer:** It performs dimensionality reduction along the depth axis while preserving spatial dimensions, as described in Equation 11.


(11) $$X_{3}=\text{ReLU }\left(W_{2}*X_{2}+b_{2}\right)$$

- Filters: *W*_2_ with shape 64 × 1 × 1 × 64.
- Output Shape: $X_{3}\in {\mathbb{R}}^{54\times 54\times 64}$ .

**4. Third Convolution Layer:** It captures more complex features through deeper, larger filters, as shown in Equation 12.


(12) $$X_{4}=\text{ReLU }\left(W_{3}*X_{3}+b_{3}\right)$$

- Filters: *W*_3_ with shape 192 × 3 × 3 × 64
- Output Shape: $X_{4}\in {\mathbb{R}}^{54\times 54\times 192}$ .

**5. Second Max-Pooling Layer:** It further reduces spatial dimensions to focus on the most prominent features, as shown in Equation 13.


(13) $$X_{5}=\text{MaxPool }\left(X_{4},\left(3,3\right),\text{ stride }=2\right)$$

- Output Shape: $X_{5}\in {\mathbb{R}}^{26\times 26\times 192}$ .

#### 3.4.2 Inception modules

These modules simultaneously process input with multiple filters of different sizes to capture features at various scales. They further concatenate the outputs of different paths to increase the richness of the feature representation. The generic form of the Inception block is presented in the following, as shown in Equation 14.


(14) $$X_{\text{out}\;}=\left[X_{\text{path}1\;}\|X_{\text{path}2\;}\|X_{\text{path}3\;}\|X_{\text{path}\;4}\right]$$

**1. Path 1: Single 1x1 Convolution:** It reduces the depth, making the network computationally efficient while learning localized features, as shown in Equation 15.


(15) $$X_{\text{path}1\;}=\text{ReLU }\left(W_{1\times 1}^{\left(1\right)}*X_{\text{in}}+b_{1\times 1}^{\left(1\right)}\right)$$

**2. Path 2: 1×1 Convolution followed by 3x3 Convolution:** It first reduces depth, then applies 3 × 3 filters to capture medium-sized features, as described in Equation 16.


(16) $$X_{\text{path}2}=\text{ReLU }\left(W_{3\times 3}^{\left(2\right)}*\text{ReLU }\left(W_{1\times 1}^{\left(2\right)}*X_{\text{in}}+b_{1\times 1}^{\left(2\right)}\right)+b_{3\times 3}^{\left(2\right)}\right)$$

3. **Path 3: 1x1 Convolution followed by 5x5 Convolution:** It is similar to Path 2 but uses 5 × 5 filters to capture larger features, as stated in Equation 17.


(17) $$X_{\text{path}3}=\text{ReLU }\left(W_{5\times 5}^{\left(3\right)}*\text{ReLU }\left(W_{1\times 1}^{\left(3\right)}*X_{\text{in}}+b_{1\times 1}^{\left(3\right)}\right)+b_{5\times 5}^{\left(3\right)}\right)$$

**4. Path 4: 3x3 Max Pooling followed by 1x1 Convolution:** It adds local spatial information via pooling and reduces depth using 1 × 1 convolution, as described in Equation 18.


(18) $$X_{\text{path}4}=\text{ReLU }\left(W_{1\times 1}^{\left(4\right)}*\text{MaxPool }\left(X_{\text{in}},\left(3,3\right)\right)+b_{1\times 1}^{\left(4\right)}\right)$$

#### 3.4.3 Auxiliary classifiers

In these classifiers, auxiliary loss improves gradient flow and prevents vanishing gradients in deep networks by adding auxiliary outputs contributing to total loss during training. On the other hand, regularization acts as a form of regularization, making the network more robust. These auxiliary operations are described below.

1. **First Auxiliary Classifier:** It provides an additional gradient path to stabilize the training. The calculation of the first auxiliary classifier is demonstrated in Equations 19–21.


(19) $$X_{\text{aux}1}^{\left(1\right)}=\text{ReLU }\left(W_{1\times 1}^{\left(\text{aux}1\right)}*\text{AvgPool }(X,5\times 5)\right)$$

(20) $$X_{\text{aux}1}^{\left(2\right)}=\text{ReLU }\left(W_{\text{aux}}^{\left(1\right)}\cdot \text{Flatten }\left(X_{\text{aux}1}^{\left(1\right)}\right)\right)+b$$

(21) $$X_{\text{aux}1}=\text{Dropout }\left(X_{\text{aux}1}^{\left(2\right)}\right)$$

**2. Second Auxiliary Classifier:** It assists in improving convergence speed and model performance by backpropagating additional supervisory signals. The computation of the first auxiliary classifier is demonstrated in Equations 22–24.


(22) $$X_{\text{aux}2}^{\left(1\right)}=\text{ReLU }\left(W_{1\times 1}^{\left(\text{aux }2\right)}*\text{AvgPool }(X,\left(5,5\right))\right)+b_{\text{aux}\;2}$$

(23) $$X_{\text{aux}2}^{\left(2\right)}=\text{ReLU }\left(W_{\text{aux}}^{\left(2\right)}\cdot \text{Flatten }\left(X_{\text{aux}\;2}^{\left(1\right)}\right)\right)+b_{\text{dense}\;}^{\left(\text{aux}\;2\right)}$$

(24) $$X_{\text{aux}2}=\text{Dropout }\left(X_{\text{aux}2}^{\left(2\right)}\right)$$

#### 3.4.4 Final layers

These layers comprise three main functions. First, Global Average Pooling reduces each feature map to a single value, allowing the network to output predictions independently of spatial dimensions. Second, dropout prevents overfitting by randomly omitting neurons during training. Third, Softmax produces a probability distribution over classes, enabling classification, both layers are defined by Equations 25 and 26.


(25) $$X_{\text{final}\;}=\text{Dropout }\left(\text{GlobalAveragePooling}\;\left(X_{\text{final}\_\text{block}\;}\right)\right)$$

(26) $$Y=\text{Softmax }\left(W_{\text{out}}\cdot X_{\text{final}\;}+b_{\text{out}\;}\right)$$

### 3.5 Federated learning process

The FL process is used to ensure robust data security by retaining MRI data locally at individual client stations, such as hospitals or imaging centers, eliminating the need for raw data transfer to a centralized server. In an FL setup, multiple clients train their models locally on their own data and then aggregate them on a central server. Each station independently trains a local model on its dataset using a standardized architecture, such as GoogLeNet, ensuring that data remain within its original boundaries. Upon completion of local training, only model updates, including weights and gradients, are shared with the central federated server. These updates do not contain raw data, significantly mitigating the risk of data exposure. The federated server aggregates these updates from all participating stations using techniques such as Federated Averaging, enabling the construction of a global model that leverages insights from diverse datasets without directly accessing sensitive patient information. This approach facilitates collaborative learning while maintaining strict data privacy and security standards. The FL process is described in the following.

#### 3.5.1 Local training on client *i*

Each client *i* performs local training with its dataset *D_i_* using the GoogLeNet architecture. Local training aims to optimize the model parameters *θ_i_* based on the client’s data, as defined in Equation 27.

1. Local Training Objective


(27) $$\theta_{i}^{'}=\theta_{i}-\eta \nabla_{\theta }L_{i}\left(\theta_{i};D_{i}\right)$$

where

*θ_i_*: Local model parameters on client *i*.

*η*: Learning rate.

∇*_θ_L_i_* (*θ_i_*; *D_i_*): Gradient of the loss function *L_i_* with respect to the model parameters *θ_i_* computed on dataset *D_i_*

$\theta_{i}^{'}$ : Updated model parameters after one training iteration.

2. Loss Function: For classification tasks, the loss function *L_i_* (*θ_i_*; *D_i_*) could be the cross-entropy loss is defined in Equation 28:


(28) $$L_{i}\left(\theta_{i};D_{i}\right)=-\frac{1}{\left|D_{i}\right|}\sum_{\left(x_{j},y_{j}\right)\in D_{i}}\left[y_{j}\text{ log }\left(\frac{e^{f\left(x_{j};\theta_{i}\right)}}{\sum_{k}e^{f\left(x_{j};\theta_{i},k\right)}}\right)\right]$$

where *f* (*x_j_*;*θ_i_*) is the predicted logit for input *x_j_* with parameters *θ_i_*, and *y_j_* is the true label.

3. Gradient Computation: The gradient ∇*_θ_L_i_* (*θ_i_*; *D_i_*) is computed using backpropagation through the GoogLeNet model. It involves computing derivatives of the loss with respect to each parameter in the network.

4. Update Rule: The parameter update involves subtracting the product of the learning rate *η* and the gradient from the current parameters.

#### 3.5.2 Model aggregation at the central server

Once local training is completed, each client sends its updated model parameters to a central server, which aggregates these parameters to form a global model using Equation 29.

1. Federated Averaging


(29) $$\theta =\frac{\sum_{i=1}^{N}\left|D_{i}\right|}{\left|D\right|}\theta_{i}^{'}$$

where

*θ*: Aggregated global model parameters.

*N*: Number of clients.

|*D_i_* |: Size of the dataset on client *i*.

|*D*|: Total data size across all clients.

2. Weight Averaging: The global model *θ* is calculated as a weighted average of the local models $\theta_{i}^{'}$ , where the weights are proportional to the sizes of the datasets |*D_i_* | on each client. This ensures that clients with larger datasets have a more significant influence on the global model.

3. Impact of Data Size: The aggregation process accounts for the size of each client’s data. If |*D_i_* | varies significantly between clients, the averaging adjusts the global model better to reflect the contributions of clients with larger datasets.

### 3.6 Explainable AI for model interpretation

In proposed architecture we incorporated two methods Grad-CAM and Saliency Maps for explainability evaluation.

#### 3.6.1 Gradient-weighted class activation mapping

DL models, particularly CNNs, have exceptionally succeeded in applications such as image classification, object detection, and medical diagnosis (21). However, their black-box nature often limits their trustworthiness in critical domains such as brain tumor classification using MRI images. Grad-CAM addresses this challenge by providing interpretable visual explanations of model predictions, bridging the gap between accuracy and transparency. Grad-CAM extends the CAM technique to any CNN architecture, using gradients flowing from the output layer to intermediate convolutional layers to identify the spatial regions most relevant to a given class prediction. These regions are visualized as heatmaps overlaying the input image, pinpointing features such as tumor boundaries or abnormal tissue patterns critical for diagnosis. Grad-CAM requires no architectural modifications, works seamlessly with pre-trained models like GoogLeNet, and emphasizes meaningful contributions using ReLU activation to focus on positive influences. By offering class-specific, spatially precise, and gradient-driven visualizations, Grad-CAM enhances the interpretability of AI predictions, building trust and helping clinicians verify model decisions while uncovering valuable insights in medical data, making it an indispensable tool in AI-driven healthcare. Grad-CAM generates visual explanations by highlighting image regions that influence the prediction of the model for a specific class.

**Activations and Gradients**

- Let $A^{k}\in {\mathbb{R}}^{u\times v}$ represent the activation map of the *k*-th channel from the target convolutional layer.
- Let *α^k^* be the weight corresponding to the *k*-th channel, computed by Equation 30 as:

(30) $$\alpha^{k}=\frac{1}{u\times v}\sum_{i=1}^{u}\sum_{j=1}^{v}\frac{\partial y^{c}}{\partial {\text{A}}_{ij}^{k}}$$

Where

-
$y^{c}$ is the score for class*c* (logit or pre-softmax output).
-
$\frac{\partial y^{c}}{\partial {\text{A}}_{ij}^{k}}$ is the gradient of $y^{c}$ w.r.t. the activation map $A^{k}$ .

**Weighted Combination** Combine the activation maps **A** ^*k*^ using the weights *α^k^* to generate the class activation map, as formulated in Equation 31.


(31) $$\text{CAM }(i,j)=\text{ReLU }\left(\sum_{k}\alpha^{k}A_{ij}^{k}\right)$$

where *ReLU* ensures only positive influences contribute.

#### 3.6.2 Normalization

Normalize *CAM* for better visualization defined in Equation 32:


(32) $${\text{CAM}}_{\text{norm}}(i,j)=\frac{\text{CAM}\;(i,j)-\min\;(\text{CAM})}{\max\;(\text{CAM})-\min\;(\text{CAM})}$$

This maps the values to [0,1] .

#### 3.6.3 Saliency maps

Saliency maps offer a solution by providing interpretability, allowing us to visualize which regions in the input image have the greatest influence on the model’s prediction. Saliency maps are used to interpret how CNNs arrive at their predictions by highlighting important pixels or regions that affect the output of a specific class. Saliency maps visualize the importance of each pixel in the input image by calculating the gradient of the output with respect to the input, identifying which regions of the input were most influential in determining the model’s decision.

The saliency map for class *c* can be mathematically defined in Equation 33:


(33) $$S^{c}{\left(\text{x}\right)}_{i}=\left|\frac{\partial y^{c}}{\partial x_{i}}\right|$$

where:

-
$S^{c}{\left(\text{x}\right)}_{i}$ is the saliency map at pixel i ,
-
$\frac{\partial y^{c}}{\partial x_{i}}$ is the gradient of the class score $y^{c}$ with respect to the pixel $x_{i}$ in the input image.

These gradients offer an explanation of the importance of each pixel in the decision-making process of the model by showing how variations in each pixel impact the sensitivity of the model’s output for class *c*.

**Saliency Map Calculation**

The saliency map is generated by computing the gradient of the class score with respect to each pixel in the input. The gradient is calculated by backpropagation, which involves the following steps:

1. To calculate the class score *y^c^* for a given input **x**, perform a forward pass.
2. Determine the gradient of *y^c^* for every *x_i_* pixel in the input image.
3. Take the absolute value of these gradients to generate the saliency map.

Thus, the saliency map can be computed in Equation 34:


(34) $$S^{c}\left(x\right)=\left[\left|\frac{\partial y^{c}}{\partial x_{1}}\right|,\left|\frac{\partial y^{c}}{\partial x_{2}}\right|,\ldots ,\left|\frac{\partial y^{c}}{\partial x_{d}}\right|\right]$$

This vector can be reshaped into a heatmap for visualization, where regions with higher values indicate greater importance in the decision-making process of the model.

**Weighted Aggregation of Gradients** In sophisticated approaches, such as integrated gradients, the gradient values are aggregated with a range of inputs that span from a baseline to the actual input. This process helps to capture the total influence of the input on the model decision-making process. The saliency map for integrated gradients is defined in Equation 35:


(35) $$S_{\text{IG}}^{c}{\left(\text{x}\right)}_{i}=\left(x_{i}-{\tilde{x}}_{i}\right)\int_{\alpha =0}^{1}\frac{\partial y^{c}\left(x^{\alpha }\right)}{\partial x_{i}}d\alpha$$

where:

-
${\text{x}}^{\alpha }$ is a path from the baseline input ${\tilde{x}}_{i}$ to the actual input $x_{i}$ ,
-
$\frac{\partial y^{c}\left(x^{\alpha }\right)}{\partial x_{i}}$ is the gradient at a point along the path,
- The integral accumulates the gradient along the path from ${\tilde{x}}_{i}$ to $x_{i}$ .

**Normalization** To facilitate interpretation, normalization of the saliency map to the range allows the most significant regions to be represented and makes it easier to interpret. The normalization step is illustrated in Equation 36:


(36) $$S_{\text{norm}\;}^{c}{\left(\text{x}\right)}_{i}=\frac{S^{c}{\left(\text{x}\right)}_{i}-\min\;\left(S^{c}\left(\text{x}\right)\right)}{\max\;\left(S^{c}\left(\text{x}\right)\right)-\min\;\left(S^{c}\left(\text{x}\right)\right)}$$

The saliency map is normalized by using the interval [0,1] which makes it easier and also enhances the visibility of regions on the input image’s classification.

## 4 Experiments and performance evaluation

This section elaborates on the configuration of the experimental setup and a brief discussion of the results.

### 4.1 Implementation platform

The architecture was developed in Google Colab Pro, a cloud-based environment offering substantial computational capabilities, ideal for efficient machine learning applications. The GoogLeNet architecture selected as the base model is widely recognized as a deep CNN architecture employed through the PyTorch framework. Google Colab Pro, with its advanced hardware configuration featuring the NVIDIA L4 GPU, delivered the necessary computational power and speed essential to efficiently handle the training and evaluation of MRI image datasets. This configuration enabled efficient execution of the FL process, supporting distributed training across several nodes without performance degradation, making it an optimal choice to tackle the resource-intensive challenges of MRI image analysis. The proposed model is trained with optimal hyperparameters selected through extensive experiments. **Table 1** presents the utilized hyperparameters.

**Table 1:** The utilized hyperparameters.

Hyperparameters	Values/Description
Image size	224 x 224
Num of Clients	10
No of rounds	50
Batch size	128
Epochs per client	3
Learning Rate	0.002
Optimizer	SGD
Momentum	0.0
Ema momentum	0.99
Base Model	Pre-trained GoogLeNet
Device Configuration	CUDA (GPU)

### 4.2 Performance assessment parameters

A number of evaluation metrics were defined to analyze the performance of the proposed architecture. Details of the general evaluation metrics are described below.

#### 4.2.1 General evaluation metrics

1. **Accuracy** measures the proportion of correctly classified samples out of the total samples. In brain tumor classification, accuracy indicates the level of model correctness in the identification of the presence or absence of tumors in all samples.


(37) $$\;\text{Accuracy}\;=\frac{TP+TN}{TP+TN+FP+FN}$$

2. **Precision** measures the proportion of correctly predicted positive samples out of the total predicted positive samples. The precision reflects the accuracy of the model in predicting tumor cases. High precision means that in most cases the model predicted as tumors is indeed a tumor.


(38) $$\;\text{Precision}\;=\frac{TP}{TP+FP}$$

3. **Recall** or sensitivity measures the proportion of correctly predicted positive samples out of the actual positive samples. Recall indicates how well the model identifies actual tumor cases. High recall means that the model detects most of the tumor cases, minimizing false negatives.


(39) $$\;\text{Recall}\;=\frac{TP}{TP+FN}$$

4. **F1 Score** is the harmonic mean of precision and recall, which helps to create a balance between the two metrics, especially in the case of an imbalanced class distribution.


(40) $$\;\text{F}1\;\text{Score}\;=2\times \frac{\;\text{Precision}\;\times \;\text{Recall}\;}{\;\text{Precision}\;+\;\text{Recall}\;}$$

5. **Confusion Matrix** is a table that summarizes the performance of a classification model by comparing actual and predicted labels.


(41) $$\;\text{Confusion}\;\text{Matrix}\;=\left[\begin{array}{cccc} C_{11} & C_{12} & \ldots & C_{1n}\\ C_{21} & C_{22} & \ldots & C_{2n}\\ \vdots & \vdots & \ddots & \vdots \\ C_{n1} & C_{n2} & \ldots & C_{nn} \end{array}\right]$$

6. **Classification Report** provides a detailed overview of the model’s performance for each class, which is particularly useful in medical applications like brain tumor classification, where the distinction between classes (different tumor types) is critical.

#### 4.2.2 Explainability evaluation metrics

1. **Heatmap Visualization:** This technique highlights the key regions of an image that a model uses to inform its conclusions. Cool hues like blue and green suggest that these areas are less significant, while warm hues like red and orange suggest that they are more significant.

2. **Grad-CAM Visualization:** This method allows us to observe how the various regions of an image contribute to the prediction by displaying the key areas in a heatmap that are most pertinent to a model’s prediction of a particular class. The mathematical formulation of the Grad-CAM visualization is presented in Equation 42.


(42) $$\alpha_{k}^{c}=\frac{1}{Z}\sum_{i}w_{k}^{c}\cdot \text{ReLU }\left(A_{i}^{c}\right)$$

where

-
$\alpha_{k}^{c}$ is the weight for feature map *k* and class *c*.
-
$w_{k}^{c}$ represents the gradient of the class score *c* with respect to the feature map *k*.
-
$A_{i}^{c}\;$ is the activation of the unit *i* in the feature map *k*.
- *Z* is the normalization factor (sum of all activations).

**3. Saliency Map Visualization:** Saliency maps aim to identify which pixels in an input image are most critical for the model’s output. They identify the pixels that have the greatest influence on the prediction score, enabling a detailed understanding of the importance at the pixel level. The calculation of saliency is based on the gradient of the output in relation to the input as shown in Equation 43:


(43) $$S_{i}=\left|\frac{\partial y}{\partial x_{i}}\right|$$

- *S_i_* is the saliency score for pixel *i*.
- *y* represents the output of the model (prediction score).
- *x_i_* is the intensity value of the pixel *i*.

### 4.3 Discussion on experimental outcomes

**Figure 3:**
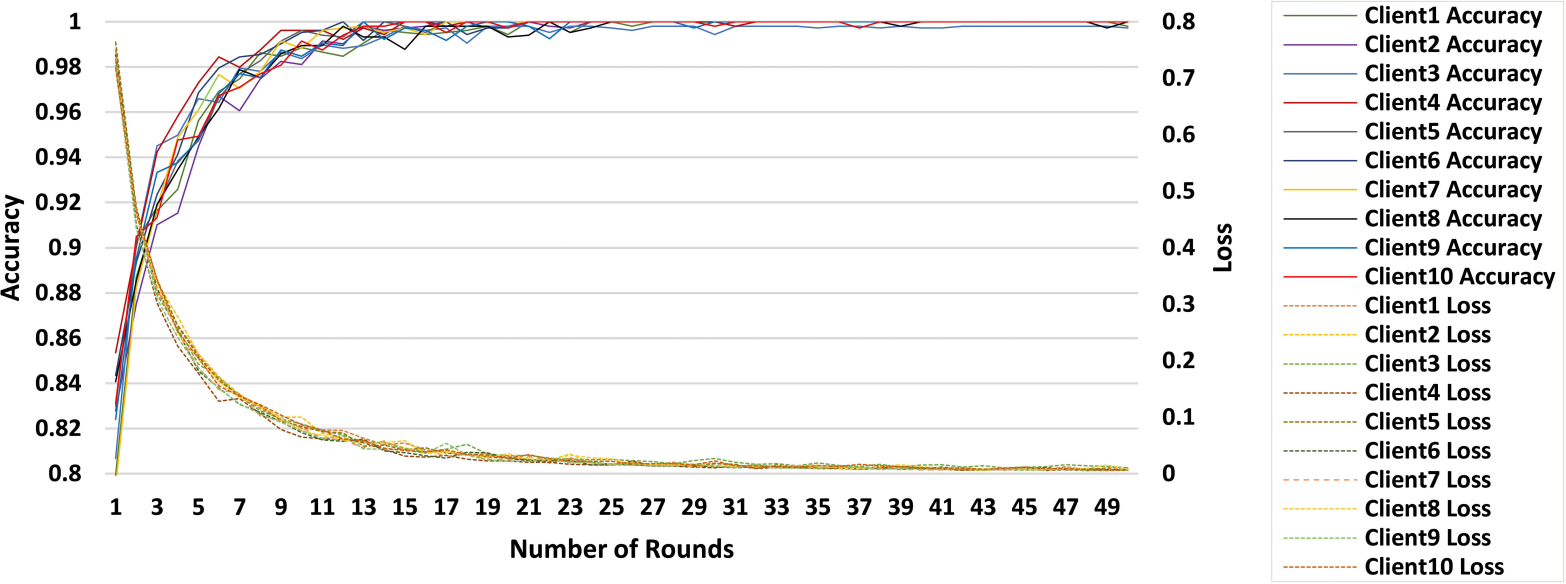
Training and loss curves for all clients.

**Figure 4:**
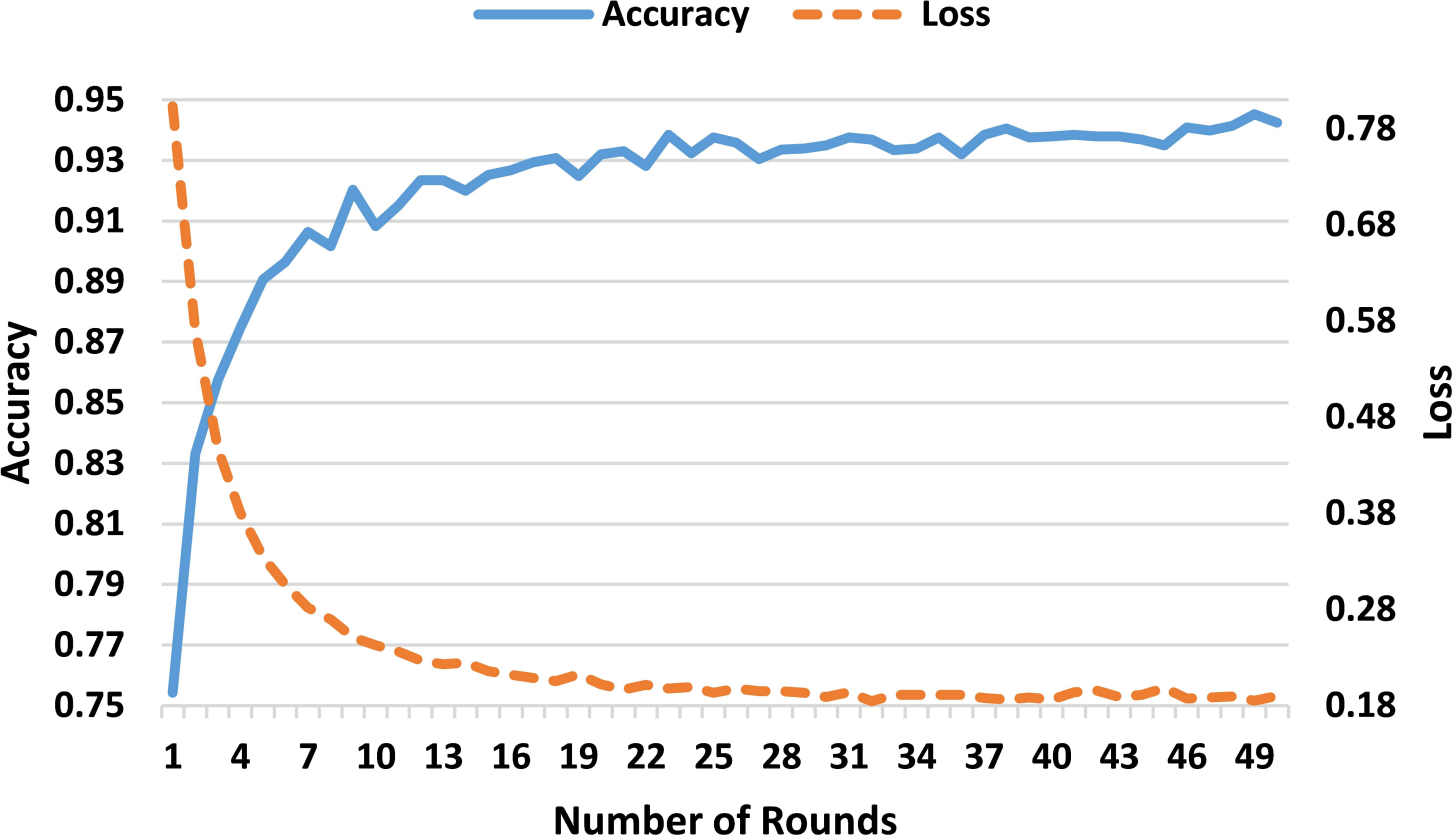
Training and loss curves of the global model.

The proposed FL-based brain tumor classification approach was implemented using GoogLeNet as the base model. The pre-trained GoogLeNet exhibits superior performance in image classification tasks. Its inception architecture efficiently handled large-scale image data by reducing the number of parameters and computational requirements, making it suitable for medical imaging with an image size of 224x224. In the FL process, 10 clients participated in the local training with a batch size of 128, using a learning rate of 0.002 to balance learning speed and stability. The training process used the stochastic gradient descent (SGD) optimizer with a momentum of 0.0 to avoid overshooting during optimization. Additionally, an Exponential Moving Average (EMA) momentum of 0.99 was applied, which smoothed the model updates over time, leading to a more stable convergence of the global model. Each client trained for 3 epochs per round and over 50 communication rounds, and the client models were aggregated to update the global model, achieving privacy-preserving training while maintaining high accuracy and computational efficiency. **Figures 3** and **4** represent the training and loss curves of the local model and globe, respectively.

The performance analysis of each individual client is summarized in **Table 2**, which details the progression of the accuracy of each client in 10 rounds. Starting with accuracies between 94% and 97% after 5 rounds, clients steadily approach 100% by the 25*th* round, with only minor fluctuations observed afterward. This stability reflects the effective convergence of the learning process, with near-perfect accuracy achieved by the 50*th* round, underscoring the robustness of the FL setup for accurate brain tumor classification.

**Table 2:** Performance evaluation of individual clients.

Clients	Number of Rounds									
	5	10	15	20	25	30	35	40	45	50
**Client-1**	0.9562	0.9886	0.9953	0.9944	1.0000	1.0000	1.0000	1.0000	1.0000	0.9980
**Client-2**	0.9448	0.9811	0.9972	0.9980	1.0000	1.0000	1.0000	1.0000	1.0000	1.0000
**Client-3**	0.9660	0.9836	0.9980	0.9980	0.9972	0.9944	0.9972	0.9972	0.9980	0.9972
**Client-4**	0.9730	0.9961	1.0000	1.0000	1.0000	1.0000	1.0000	1.0000	1.0000	1.0000
**Client-5**	0.9474	0.9961	0.9980	1.0000	1.0000	1.0000	1.0000	1.0000	1.0000	1.0000
**Client-6**	0.9685	0.9953	1.0000	0.9972	1.0000	1.0000	1.0000	1.0000	1.0000	1.0000
**Client-7**	0.9609	0.9883	0.9972	1.0000	1.0000	1.0000	1.0000	1.0000	1.0000	1.0000
**Client-8**	0.9490	0.9894	0.9878	0.9933	1.0000	1.0000	1.0000	1.0000	1.0000	1.0000
**Client-9**	0.9482	0.9847	0.9972	1.0000	1.0000	1.0000	1.0000	1.0000	1.0000	1.0000
**Client-10**	0.9493	0.9914	1.0000	0.9972	1.0000	0.9980	1.0000	1.0000	1.0000	1.0000

**Table 3** provides a detailed performance evaluation of the global model over 50 rounds of communication, with metrics of accuracy, precision, recall, and F1. The global model starts with an accuracy of 89.07% after 5 rounds and increases to 94.24% by the 50*th* round. Precision and recall are in the same trend, starting at 88.72% and 88.76%, respectively, and peaking at 94.05% and 94.21% in the 45*th* round. These results reflect the gradual and effective convergence of the model, confirming that the FL approach optimizes the classification performance over multiple communication rounds.

**Table 3:** Performance evaluation of the global model.

Parameters	Number of Rounds									
	5	10	15	20	25	30	35	40	45	50
**Accuracy**	0.8907	0.9082	0.9252	0.9320	0.9375	0.9349	0.9375	0.9379	0.9349	0.9424
**Precision**	0.8872	0.9156	0.9315	0.9386	0.9353	0.9344	0.9384	0.9363	0.9405	0.9374
**Recall**	0.8876	0.9165	0.9321	0.9396	0.9366	0.9355	0.9399	0.9382	0.9421	0.9389
**F1 Score**	0.8868	0.9158	0.9317	0.9390	0.9357	0.9348	0.9389	0.9369	0.9411	0.9380

**Figure 5:**
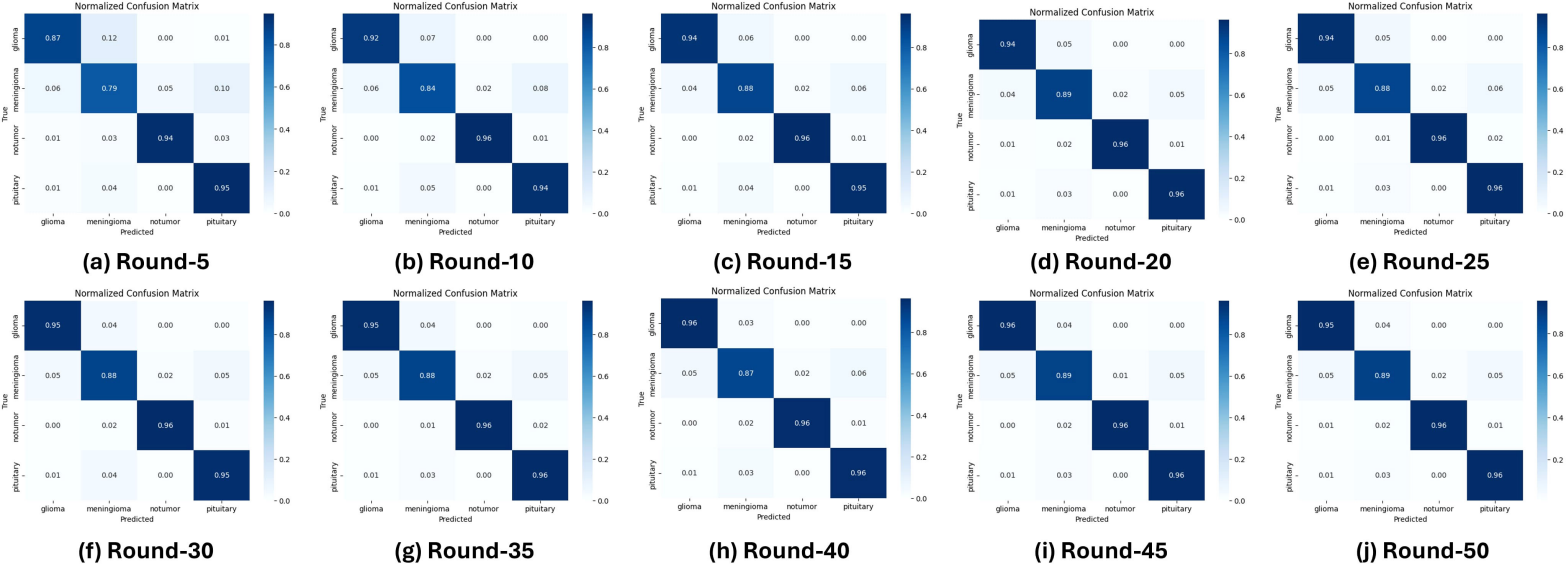
Confusion matrices for multiclass evaluation over 50 training rounds.

Performance analysis reveals that the model achieves near-perfect accuracy after 50 communication rounds, with stable convergence observed even as the number of rounds increases. In particular, **Figure 5** presents ten normalized confusion matrices that capture the classification performance for all types of glioma, meningioma, no tumor, and pituitary tumors over 50 training rounds. During training, glioma and meningioma show moderate accuracy, with frequent misclassifications between these two classes. As training progresses, the accuracy of the glioma improves substantially to 96% by round 35, while the meningioma stabilizes at 89%. The No Tumor and Pituitary classes demonstrate near-perfect accuracy from the outset, outperforming glioma and meningioma. In Round 35, the model shows strong performance, particularly for glioma, no tumor, and pituitary tumor, although there is minor confusion between glioma and meningioma.

**Figure 6:**
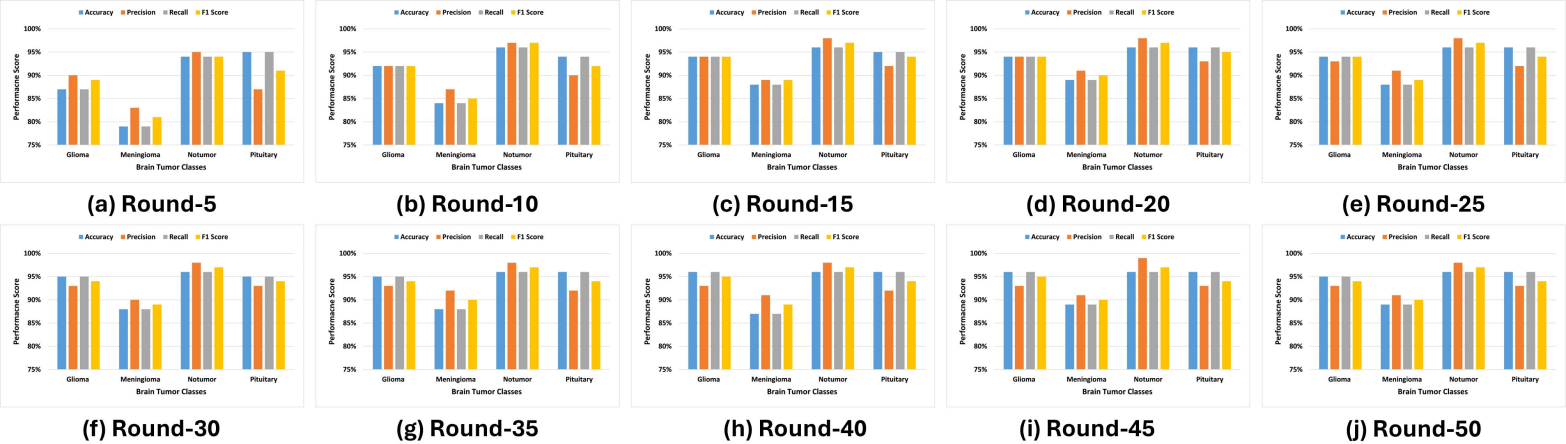
Experimental outcomes for multi-class performance.

**Figure 6** illustrates that the No Tumor class consistently achieves the highest scores in accuracy, precision, recall, and the F1 score, stabilizing near perfection by round 50. In comparison, the Meningioma class exhibits a steady upward trend in accuracy and recall, with slight variability in precision. The Pituitary class maintains robust performance throughout the training rounds, closely aligning with the No Tumor class. In contrast, while initially underperforming, the Glioma class shows significant improvements in recall and F1 score over successive rounds, reflecting effective model adaptation and learning. These results highlight the efficacy of the FL approach in improving classification performance, handling real-world variability, and addressing challenges in differentiating between specific tumor types.

**Figure 7:**
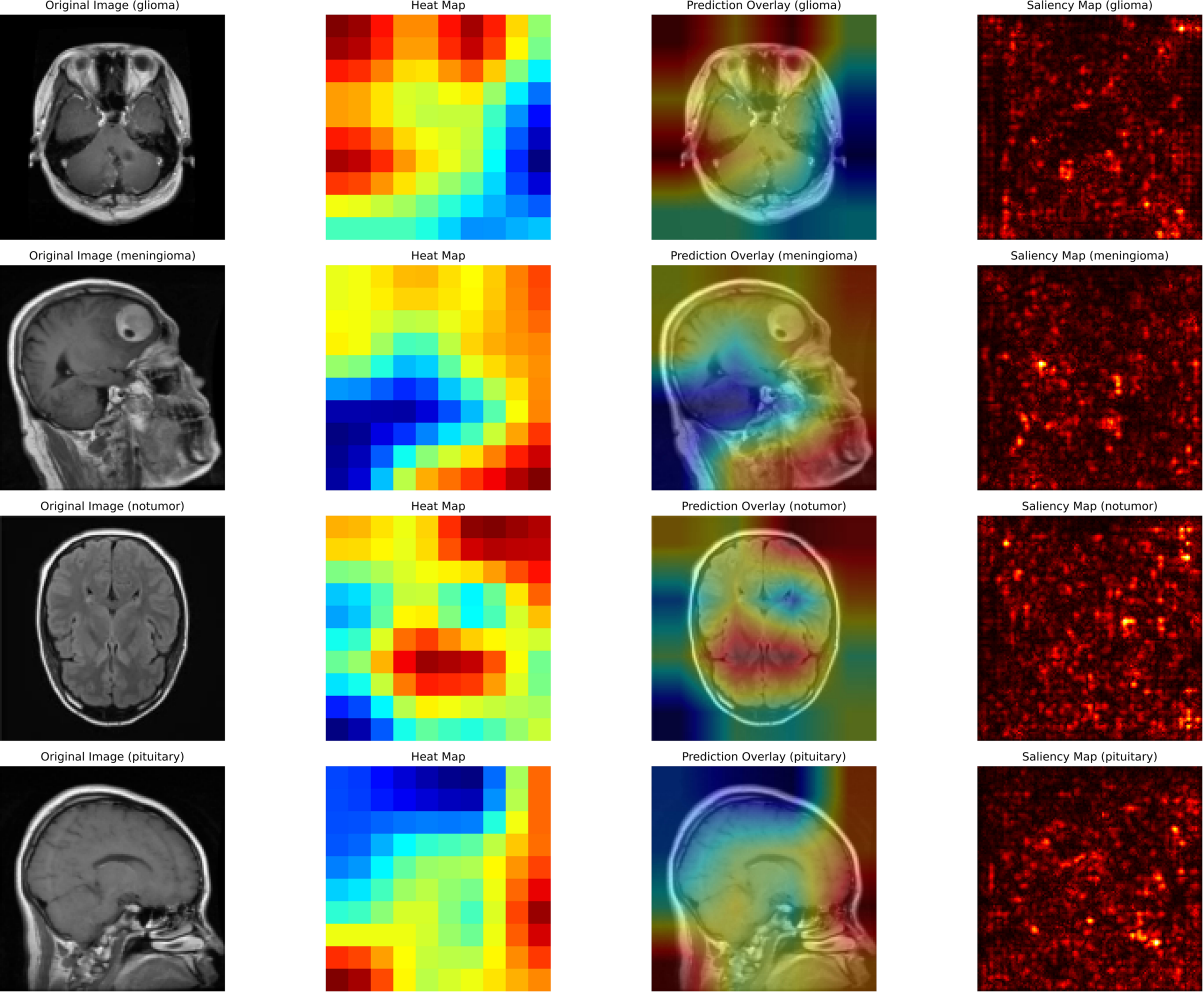
Interpretation of brain tumor classifications.

Furthermore, this study illustrates how XAI techniques are used to interpret and validate the predictions made by a deep learning model for brain tumor classification. **Figure 7** describes that each row corresponds to a specific classification scenario: glioma, meningioma, no tumor, and pituitary tumor. The columns represent different visualization techniques that provide insight into the model’s decision-making process. The first column displays the original MRI scans, which serve as input to the classification model. These scans show unique structural features of the brain, with visible abnormalities in tumor cases (glioma, meningioma, pituitary) and normal brain structures in the no-tumor case, serving as a baseline for comparison with the explainability maps.

The second column presents heat maps that highlight the regions in the MRI scans the AI model considers most critical for classification. The intensity of the color, ranging from red (high relevance) to blue (low relevance), indicates the model’s focus. For cases of glioma and meningioma, the red areas correspond closely to the visible tumor regions, demonstrating the model’s ability to locate and interpret critical features. In the no-tumor scenario, the heat map shows a dispersed focus, reflecting the absence of a specific lesion or abnormality. The pituitary tumor case illustrates the model’s focus on the pituitary gland, a relevant area for classification.

The third column overlays the heat map on the original MRI image, creating a blended visualization that helps to interpret the focus of the model relative to the actual location of the tumor. These overlays help verify whether the model is focusing on medically relevant regions. For cases of glioma and meningioma, the overlays show a strong correlation between the highlighted regions and the physical location of the tumor, which reinforces the interpretability of the model. In the no-tumor case, the overlay confirms that the model is not falsely focusing on irrelevant or random areas.

Finally, the fourth column contains saliency maps, which provide a more granular visualization by identifying the individual pixels that most influenced the model’s decision. Bright spots on these maps correspond to areas where small changes in pixel intensity would significantly impact the model’s prediction. For tumor cases, the saliency maps highlight the contours and edges of the tumor region, demonstrating the model’s ability to capture fine-grained details. This comprehensive use of XAI techniques underscores the importance of integrating explainability into medical imaging workflows, enhancing the reliability, transparency, and clinical adoption of AI-based systems for brain tumor detection.

**Table 4** presents a performance comparison with seven recent studies, demonstrating that the proposed FL scheme outperforms other models in the brain tumor classification. Our approach integrates GoogLeNet within an FL framework. GoogLeNet utilizes parallel convolutions at multiple scales within each layer, enabling the model to capture both fine and coarse details in MRI images, which is essential for detecting tumors of varying sizes and types. The combination of GoogLeNet and federated learning achieved a detection accuracy of 94%, surpassing conventional CNNs and cascade networks. Although there are few studies focusing on model interpretability, Tanvir et al. (22) employed multiple models, including CNN and InceptionV3, and integrated XAI techniques to visualize models’ latent behavior, thereby enhancing system transparency. Furthermore, they proposed Grad-CAM++ for improved model interpretability, achieving 92.31% accuracy in brain tumor classification.

**Table 4:** Performance comparison of the proposed scheme with the state-of-the art FL-based brain tumor classification model.

Reference	Proposed Technique	Dataset	Detection Score
Lakshmi et al. (23)	Inception-V3	3064 MRI Images	89%
Jiang et al. (24)	Convolutional Neural Network	MICCAI BRATS2015 DATASET	86.30%
Bhanothu et al. (25)	CNN	MRI IMAGES	77.60%
Ranjbarzadeh et al. (26)	Cascade CNN	BRATS2018 DATASET	92.03%
S et al. (27)	MobileNetV2 MobileNetV3 small MobileNetV3 big	Ream word brain image dataset	92%
Akbar et al. (28)	SVM with heterogeneous feature extraction in CNN classification	BRATS2018	77.73%
Tanvir et al. (22)	CNN,RestNet50, InceptionV3,EfficientNetB0 and NASNet-Mobile with Grad CAM++	Br35H:Brain Tumor Detection 2020	92.31%
Proposed Scheme	CFLM with Grad-CAM	brain Tumor MRI dataset	94%

In summary, the proposed framework advances brain tumor classification by integrating FL with the GoogLeNet architecture, effectively addressing the challenges of scalability and computational efficiency. By leveraging a pre-trained GoogLeNet model, the system benefits from its proven image classification performance, while FL facilitates decentralized training across multiple clients, aggregating updates through Federated Averaging to construct a robust global model. This approach efficiently accommodates heterogeneous data sources without compromising accuracy. Incorporation of XAI via Grad-CAM and the saliency map enhances the interpretability of the model by visually identifying key regions in MRI images that influence predictions, supporting tumor localization. In general, the combination of FL, GoogLeNet, and XAI not only ensures high classification performance, but also fosters transparency and trust, essential for the clinical adoption of AI in healthcare.

### 4.4 Limitations

Despite the good performance, the proposed framework has few limitations. First, the fixed client configuration assumes uniform resource availability, which may not be true in real-world scenarios where clients possess varying computational capacities, potentially impacting local training efficiency. Furthermore, dynamic scenarios, such as unstable network conditions, might lead to communication delays or interruptions, therefore impacting how fast model updates can be collected. There are also privacy risks during data transfer, as adversarial attacks can leverage vulnerabilities in model update transmissions to compromise data security even in a decentralized setup. Such challenges must be overcome to further enhance resilience and reliability in diverse real-world healthcare applications.

## 5 Conclusion

The proposed scheme introduced an FL-based brain tumor classification framework incorporating the GoogLeNet architecture and explainability evaluation methods. The proposed FL approach overcomes the limitations of traditional centralized deep learning models, which require the centralization of sensitive medical data in a single entity. The proposed scheme allows collaborative training of a model while enhancing interpretability.

The proposed approach achieved a classification accuracy of approximately 94% on a dataset of 7,042 MRI images across four tumor classes. Furthermore, the designed model integrates XAI techniques, such as Grad-CAM and saliency map visualization. Grad-CAM highlights critical regions in the MRI images that contribute the most to the model’s predictions, while saliency maps further visualize these influential features. These XAI techniques provide clinicians with a clear understanding of the AI’s decision-making process, ensuring its focus aligns with clinically relevant features. Together, they improve transparency, validate model performance, and foster trust, which facilitates the seamless integration of AI into medical imaging workflows.

Future implementations of this approach could cover other medical fields and provide a scalable infrastructure for collaborative AI applications in healthcare, where data-sharing policies are strict. The proposed approach shows great value for medical imaging and artificial intelligence, providing a way to improve diagnostics in healthcare while preserving data security, interpretability, and transparency worldwide.

## Data Availability

Publicly available datasets were analyzed in this study. This data can be found here: https://www.kaggle.com/datasets/masoudnickparvar/brain-tumor-mri-dataset.
